# Cerebral Vitamin B5 (D-Pantothenic Acid) Deficiency as a Potential Cause of Metabolic Perturbation and Neurodegeneration in Huntington’s Disease

**DOI:** 10.3390/metabo9060113

**Published:** 2019-06-11

**Authors:** Stefano Patassini, Paul Begley, Jingshu Xu, Stephanie J. Church, Nina Kureishy, Suzanne J. Reid, Henry J. Waldvogel, Richard L. M. Faull, Russell G. Snell, Richard D. Unwin, Garth J. S. Cooper

**Affiliations:** 1 Centre for Advanced Discovery and Experimental Therapeutics, Division of Cardiovascular Sciences, School of Medical Sciences, Faculty of Biology, Medicine & Health, The University of Manchester, Manchester M19 9NT, UK; stefano_patas@yahoo.it (S.P.); paul.begley59@gmail.com (P.B.); jingshu.xu@hotmail.com (J.X.); stephanie.church@manchester.ac.uk (S.J.C.); kurnina@yahoo.com (N.K.); R.Unwin@manchester.ac.uk (R.D.U.); 2 School of Biological Sciences, Faculty of Science, University of Auckland, Auckland 1142, New Zealand; s.reid@auckland.ac.nz (S.J.R.); r.snell@auckland.ac.nz (R.G.S.); 3 Owlstone Medical, Cambridge Science Park, Cambridge CB4 0GJ, UK; 4 Manchester Cancer Research Centre Building, The University of Manchester, Manchester M20 4GJ, UK; 5 Centre for Brain Research, Department of Anatomy and Medical Imaging, Faculty of Medical and Health Sciences, University of Auckland, Auckland 1142, New Zealand; h.waldvogel@auckland.ac.nz (H.J.W.); rlm.faull@auckland.ac.nz (R.L.M.F.); 6 Maurice Wilkins Centre for Molecular Biodiscovery, University of Auckland, Auckland 1142, New Zealand

**Keywords:** Huntington’s disease, neurodegeneration, vitamin B5, acetyl-coenzyme A, brain energy metabolism, acetylcholine biosynthesis, metabolomics, bioinformatics, data visualisation, data analytics

## Abstract

Huntington’s disease (HD) is a neurodegenerative disorder caused by an expanded CAG repeat in exon 1 of the *HTT* gene. HD usually manifests in mid-life with loss of GABAergic projection neurons from the striatum accompanied by progressive atrophy of the putamen followed by other brain regions, but linkages between the genetics and neurodegeneration are not understood. We measured metabolic perturbations in HD-human brain in a case-control study, identifying pervasive lowering of vitamin B5, the obligatory precursor of coenzyme A (CoA) that is essential for normal intermediary metabolism. Cerebral pantothenate deficiency is a newly-identified metabolic defect in human HD that could potentially: (i) impair neuronal CoA biosynthesis; (ii) stimulate polyol-pathway activity; (iii) impair glycolysis and tricarboxylic acid cycle activity; and (iv) modify brain-urea metabolism. Pantothenate deficiency could lead to neurodegeneration/dementia in HD that might be preventable by treatment with vitamin B5.

## 1. Introduction

HD is a slowly progressive, autosomal dominant neurodegenerative disorder with broad but distinct phenotypes characterised by involuntary movements, incoordination, cognitive decline, personality changes, and psychiatric manifestations [1,2]. It is caused by an expanded unstable (CAG)n repeat at chromosome 4p16.3 in exon 1 of the *HTT* gene [3,4,5], which encodes an altered form of the huntingtin protein containing an elongated polyglutamine tract, comprising 37 or more glutamine residues [6]. Estimated costs for attributable health and social care in the UK alone are ~£200 million annually [7]. 

The neuropathological hallmark of HD is the gradual loss of medium spiny GABAergic projection neurons of the neostriatum, with slow atrophy of the caudate nucleus, putamen, and the external segment of the globus pallidus [8]. As the disease worsens, atrophy spreads throughout the brain symmetrically, with progressive loss of parenchyma from the following anatomical regions, in decreasing order of severity: cerebral white matter, thalamus, cerebral cortex, and cerebellum [8]. At the end stage, the brain is usually diffusely smaller than normal, having typically lost ~200 g (16.0%) compared with age-matched controls [9], with most damage having occurred in the dorsal striatum (putamen and caudate nucleus). The extent of brain-weight loss in end-stage HD on average is similar to that in Alzheimer’s disease (AD), ~200 g (15.7%) [10], consistent with similar wide-spread tissue damage in both. HD causes progressive loss of both neuronal and non-neuronal cells in affected brain regions [11], affecting both grey and white matter and demonstrating prominent glucose hypometabolism, as measured by brain imaging [12,13]. 

Alterations in numerous molecular processes, in the brain as well as other organs, have been implicated in the pathogenesis of HD, but how these might relate to underlying mutations in the *HTT* gene remains to be determined [14,15]. One recent line of investigation has focussed on applications of metabolite profiling in brain research, to shed new light on the pathobiology of CNS disorders, although such applications have been somewhat limited to date [16,17,18]. Perturbations leading to potentially toxigenic levels of small polar metabolites, including glucose, sorbitol, fructose, glucose-6-phosphate, and urea have now been identified in the brain in HD [9,19,20]. These findings implicate alterations in the central pathways of energy metabolism, including glycolysis, the polyol-pathway, the tricarboxylic acid (TCA) cycle, and the urea cycle/brain urea metabolism, in HD pathogenesis. 

Replication studies are required to support and extend metabolite-profiling approaches [21], whose performance in the brain have not been extensively investigated hitherto. Therefore, we have performed a replication and extension study to better characterise the performance characteristics of our approach for metabolic profiling of brain tissue, with emphasis on cases of HD and matched controls. We sought to evaluate the performance of our analytical methods and the reproducibility of results generated over time, to validate our previous metabolic profiling study [9], and to increase our ability to search for further informative metabolic perturbations. We evaluated four distinct aspects of our methodology: (1) intra-assay reproducibility of quantitative measurements; (2) metabolite stability in processed samples during residence time in the analytical machinery; (3) the accuracy of measurements of polar metabolites over a 1-year time-period; and (4) metabolite stability in human brain during long-term storage of brain samples. 

We analysed short-post-mortem-delay brain tissue from 30 cases of HD and 19 controls obtained from the New Zealand Neurological Foundation Human Brain Bank (Centre for Brain Research, University of Auckland, New Zealand) using the same criteria as in our previous study, in order to optimize processes by which tissue was collected, and to minimize variability in tissue handling [9,19]. 

We performed two independent gas chromatography-mass spectrometry (GC-MS) case-control experiments in which we analysed a total of 98 brain-tissue samples, half from the cerebellum (CB) and half from superior frontal gyrus (SFG) of 30 HD mutation-carriers and 19 matched controls. We employed comprehensive quality control replicates to measure intra-assay reproducibility, accuracy, and metabolite stability. We used our GC-MS methodology to reproducibly identify and quantitate 63 distinct polar metabolites in these brain tissues, metabolite groups included ‘glucose metabolites and pentoses’; ‘alternative fuel sources’; ‘TCA and urea-cycle and related intermediates’; ‘amino acids’; ‘nucleosides’; and ‘miscellaneous’, which included neurotransmitters. 

Interestingly, in this study, vitamin B5 (pantothenic acid), a small molecule which is the obligatory primary precursor in the biosynthetic pathway for CoA, was ranked first among all metabolites significantly changed in abundance in HD human brains, compared with controls. Vitamin B5 is an essential trace nutrient that exists in the brain at concentrations of up to 50-fold those in plasma [22]. Its utilisation in the CoA biosynthetic pathway is determined by a phosphorylation event catalysed by the enzyme pantothenate kinase (PanK), which in humans has different isoforms produced from four distinct genes, PANK1-4 [23]; mutations in one of these, PANK2, cause neurodegeneration [24,25]. Among its multitude of functions, CoA participates in shaping brain-cell architecture through its critical roles in many different biosynthetic pathways [26]. CoA is converted into acetyl-CoA by the action of pyruvate dehydrogenase [27] and provides the main substrate for the TCA cycle in all cells. Acetyl-CoA is an obligatory cofactor for ~4% of all mammalian enzymes [28], it plays key roles in control of cell growth, proliferation, and global histone acetylation, and pathways of anabolism and/or catabolism of carbohydrates, fatty acids, lipids and phospholipids, cholesterol, amino acids, water-soluble vitamins, proteins, and RNA. Furthermore, vitamin B5 participates via acetyl-CoA in the production of steroid hormones and acetylcholine in the brain [29]. 

Here, we sought to determine how vitamin B5 is physiologically distributed in the human brain, and how its CNS levels might be affected in HD-mutation carriers. We therefore employed GC-MS to quantitate vitamin B5 in 12 anatomically-distinct regions in HD and control brains. Our data demonstrate that vitamin B5 concentrations were significantly reduced in HD mutation-carriers in 8 of the 12 brain areas we analysed, and that a global decrease of the abundance of vitamin B5 is present in the HD brain. 

We conclude that vitamin B5 deficiency is a newly recognized metabolic defect in the brain of patients affected by HD. Genetically-mediated defects in the biosynthetic pathway by which vitamin B5 is converted to CoA cause neurodegeneration and dementia in humans [29], which may well be related to deficient brain levels of CoA. Therefore, our findings of pervasive lowering of vitamin B5 levels in brains affected by HD, taken together with the compelling evidence relating defective CoA biosynthesis to mechanisms of neurodegeneration in humans, indicate that vitamin B5 deficiency could lead to neurodegeneration and dementia in HD, and that these defects might be preventable by treatment with vitamin B5. 

## 2. Results

We matched subjects for age and post-mortem delay (PMD) in this case-control study (Table 1). Brain-weights were significantly greater in controls than HD mutation-carriers (Mann–Whitney U, *p* < 0.0001), consistent with the advanced neurodegeneration typically observed in late-stage cases. The HD brains studied ranged from Vonsattel neuropathological grade 0 to 4, and all cases were heterozygous for *HTT* mutations, with pathological CAG tract-lengths that ranged from 39 to 53 repeats (Supplementary Table S1). We measured the abundance of 63 metabolites in two regions (CB, SFG) from 30 cases with HD and 19 controls (Supplementary Table S1). 

**Figure 1:**
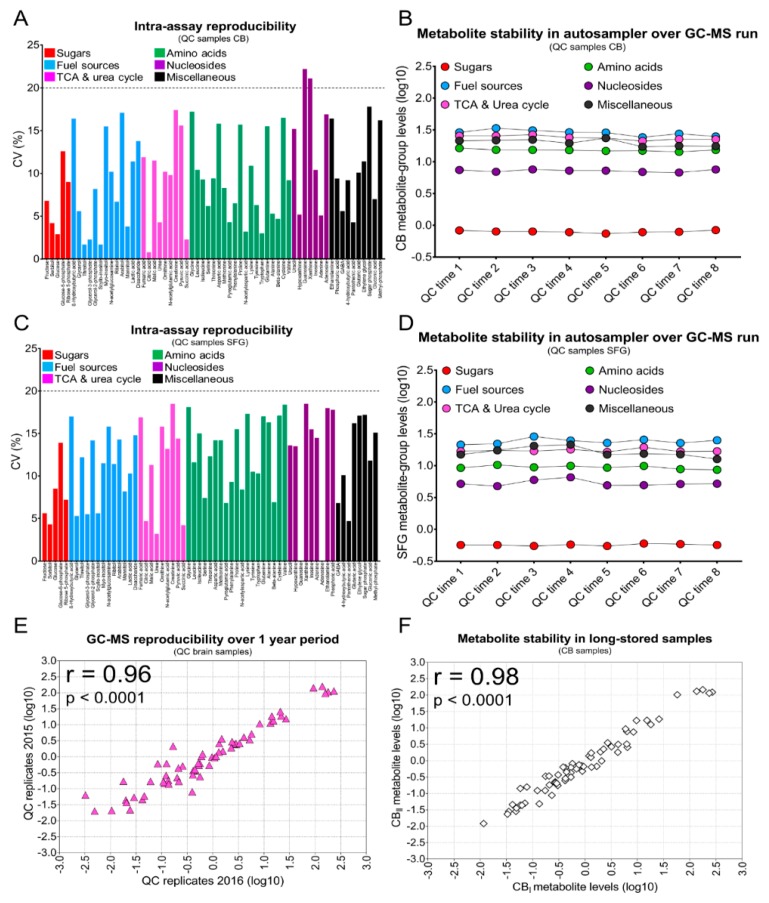
Reproducibility of the GC-MS assay and stability of metabolites during long-term storage. (**A**,**C**) Bar plots illustrate the inter-assay GC-MS reproducibility of the 63 metabolites quantified in QC samples from CB and SFG, respectively. QC replicates of pooled brain extracts were used to assess the reproducibility of measurements. For each metabolite, the coefficient of variation (%) is shown in individual brain regions. (**B**,**D**) Dot plots showing how stable the metabolite groups are in QC samples over a GC-MS sequence run of ~43 h. In this study, eight QC replicates were used for reproducibility assessment and metabolite stability investigation of CB and SFG samples. A total of 16 human brain samples homogenates were used to perform reproducibility and stability assessment. (**E**) Individual scatterplots showing the correlation between metabolites measurements in QC replicates analysed by GC-MS in 2015 (*n* = 12) and 2016 (*n* = 12). Pearson’s correlation coefficients (*r*) and corresponding *p*-values are shown at the top-left in each scatterplot panel and reported metabolite values have been log_10_-transformed. (**F**) Individual scatterplots showing the correlation between metabolites measurements in long-stored brain tissue CB biological replicates extracted in 2015 and 2016 respectively and analysed by GC-MS. Pearson’s correlation coefficients (*r*) and corresponding *p*-values are shown at the top-left in each scatterplot panel and reported metabolite values have been log10-transformed.

To have a high degree of confidence in our study, we rigorously tested the robustness of the analytical platform used and the quality of the metabolite measurements in the tissue samples analysed. First, we assessed the intra-assay reproducibility of a complete GC-MS run. Each run comprised an analytical batch of 49 metabolite extracts, eight pooled quality control (QC) sample replicates, and two saline-blank samples; these were TMS-derivatised and then analysed by GC-MS over ~43 h/run. In the QC-pool replicates (*n* = 8) from CB extracts, almost all metabolites (97%) had %CV values of <20%, indicating excellent intra-assay reproducibility (Figure 1A). We obtained equivalent results for pooled extracts from SFG-derived QC samples (Figure 1C). We also measured the variability of mean abundances in metabolite-groups using QC-pooled replicates from CB, during residence time in the GC autosampler (~43 h). Groups analysed included amino acids, sugars, nucleosides, and TCA-cycle/urea-cycle-related metabolites, all of which had CV values of <10%; other metabolite-groups had CV values of <15%. These findings show that the GC run-time delay did not significantly influence metabolite stability (Figure 1B,D). To assess the accuracy of our GC-MS platform over the period of the entire study, we calculated the correlation coefficients generated by comparing the metabolite abundances of processed QC replicates analysed by GC-MS immediately, and one year later. We observed strong correlation between QC samples, showing the excellent stability of the analytical platform across this time (*r* = 0.96; *p* < 0.0001; Figure 1E). Lastly, we evaluated the stability of metabolites in human brain-tissue samples during long-term storage. To investigate this, metabolites were extracted on different occasions using brain-tissue replicates as starting material; extracts were then batch-analysed by GC-MS to compare metabolite abundances during long-term storage. The small variability observed in correlation analyses, even for low-abundance metabolites, indicates that sample storage has a minimal effect on the overall quality of our GC-MS measurements (*r* = 0.98; *p* < 0.0001; Figure 1F). Taken together, the minimal variability introduced by the sample-processing procedures and the analytical method applied support the excellent quality of the dataset produced here. 

**Figure 2:**
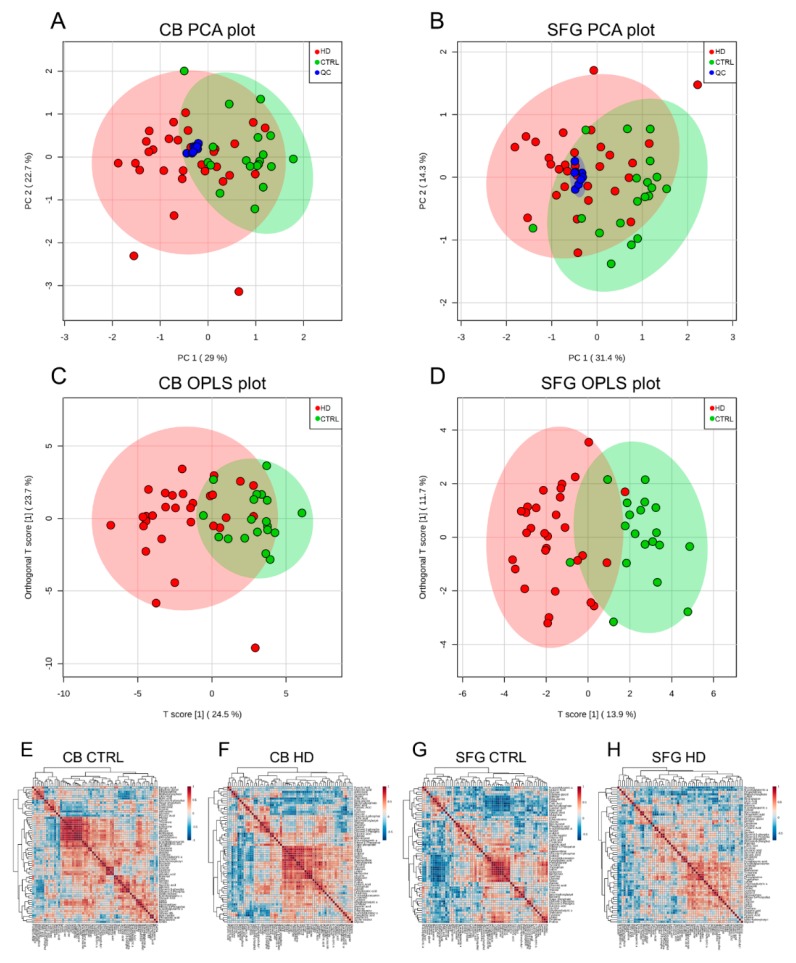
Shown are PCA, OPLS-DA, and hierarchical correlation heatmaps of CB and SFG brain samples analysed by GC-MS. (**A**,**B**) PCA plots showing limited class separation in CB and SFG brain areas between HD cases (red circles) and controls (green circles). In CB samples, the first principal component (PC 1) explains 29.0% of the total variance in the subjects examined, the second (PC2) an additional 22.7%. In SFG the PC1 represents 31.4% of the total variance whereas PC2 contributes 14.3%. The different conditions (HD vs. controls) can only be partially separated in the PCA plots. The compact clustering observed between the QC replicates (blue circle) supports the high degree of reproducibility of the GC-MS assay used here. (**C**,**D**) OPLS-DA model results for CB and SGF brain regions in HD (red circles) and controls (blue circles). Limited separation is observed between HD cases and controls in both brain regions analysed by GC-MS. Coloured ellipsoids around each group represent 95% confidence intervals. (**E**–**H**) Hierarchical correlation heatmaps illustrating the differential clustering and metabolite abundance co-regulation in CB (left panels **E**,**F**) and SFG (right panels **G**,**H**) in controls and HD mutation-carriers. Metabolites were clustered with a Euclidean distance algorithm and differences between co-regulation of metabolite abundances were investigated by Pearson correlation analyses. Pearson’s correlation coefficient (*r*) values are visually depicted in the colour gradient scales on the right side of each heatmap.

We employed this GC-MS methodology to explore the effects of the HD mutation on human brain metabolite abundances. Multivariate principal-component analysis (PCA) analyses of CB and SFG samples (*n* = 49: 19 controls, 30 HD) and metabolites (63 in total) were performed (Figure 2A,B). On the PCA plot for CB analyses, HD mutation-carriers were poorly separated from the controls with a minor overlap between case and control groups (PC1 = 29%, PC2 = 22.7%; Figure 2A). Similarly, a modest separation effect was evident in the PCA plot of SFG metabolites (PC1 = 31.4%, PC2 = 14.3%; Figure 2B). We followed this analysis by Orthogonal Projections to Latent Structures Discriminant Analysis (OPLS-DA) modelling using one orthogonal component and one predictive value, with validation by permutation analysis (Figure 2C,D). The OPLS-DA score plot and the p (corr) s-plot for CB-derived data shows the clustering of HD and control groups, and the individual contribution of metabolites in separating the two groups, respectively (Figure 2C; Supplementary Figure S2A). With a weighted sum absolute regression coefficients analysis on the CB OPLS-DA model, we measured the top 10 metabolites responsible for separation in CB samples, which included in descending order: vitamin B5, sorbitol, scyllo-inositol, glucose, adenine, fructose, malic acid, glutaric acid, urea, and citric acid (Supplementary Data S1). The SFG OPLS-DA model showed a clustering pattern similar to those obtained from CB samples (Figure 2D; Supplementary Figure S2B), however several of the ten metabolites contributing the most to group separation differed, namely: vitamin B5, glycerol-2-phosphate, urea, fructose, sorbitol, glycerol-3-phosphate, N-acetylglutamic acid, scyllo-inositol, mannitol and cysteine. To further investigate the effect of HD mutation on metabolite regulation, an exploratory pair-wise correlation analysis with hierarchical clustering was performed for CB and SFG, and the results of each group compared. Different correlation and clustering patterns were observed between controls and HD cases in both CB and SFG samples, indicative of dysfunctional metabolic regulation in specific brain regions in HD (Figure 2E–H). In addition, the correlation analyses revealed that metabolites participating as products of amino-acid metabolism and intermediates of the polyol-pathway, were among the major contributors to the clustering pattern obtained from CB and SFG samples in HD subjects (Figure 2E–H). The list of the metabolites used to generate PCA plots, OPLS-DA models, and correlation analyses is shown in Supplementary Table S2. 

**Figure 3:**
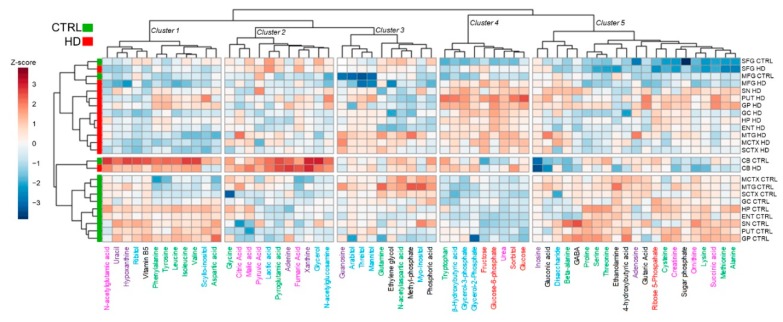
Analysis by hierarchical clustering of metabolite abundances in control and HD brain. This shows substantial separation between controls (green) and HD cases (red) across brain regions. Of interest, the most significant clustering is present in cluster 4 (*p* = 7.57 × 10^−5^, FDR-corr), which is primarily enriched by categories of metabolites as alternative fuel sources, sugars, and urea. The Euclidean distance method was used for hierarchical clustering; values are expressed as Z-scores and visually represented as a colour-gradient scale.

To gain insights into the global metabolite remodelling caused by pathogenic *HTT* mutation in the human brain, we performed HC analyses, combining the current dataset with others acquired in parallel by our group, with the same optimised profiling GC-MS workflow (Figure 3) [9]. Unsupervised HC separated HD and control subjects in three main clusters, indicating that a robust metabolite dysregulation is occurring in distinctive brain areas upon presence of HD mutation. Notably CB, MTG, and SFG, three regions relatively spared until advanced HD stages, showed the most similar clustering profile between HD and control subjects (Figure 3). Based on the metabolite abundance profile instead, five major clusters were obtained. The most relevant clusters were enriched in metabolites of galactose metabolism (cluster 4, *p* = 7.57 × 10^−5^, FDR-corr), citric acid (TCA) cycle intermediates (cluster 2, *p* = 1.35 × 10^−4^, FDR-corr), and beta-alanine metabolism (cluster 1, *p* = 6.26 × 10^−4^, FDR-corr). 

**Figure 4:**
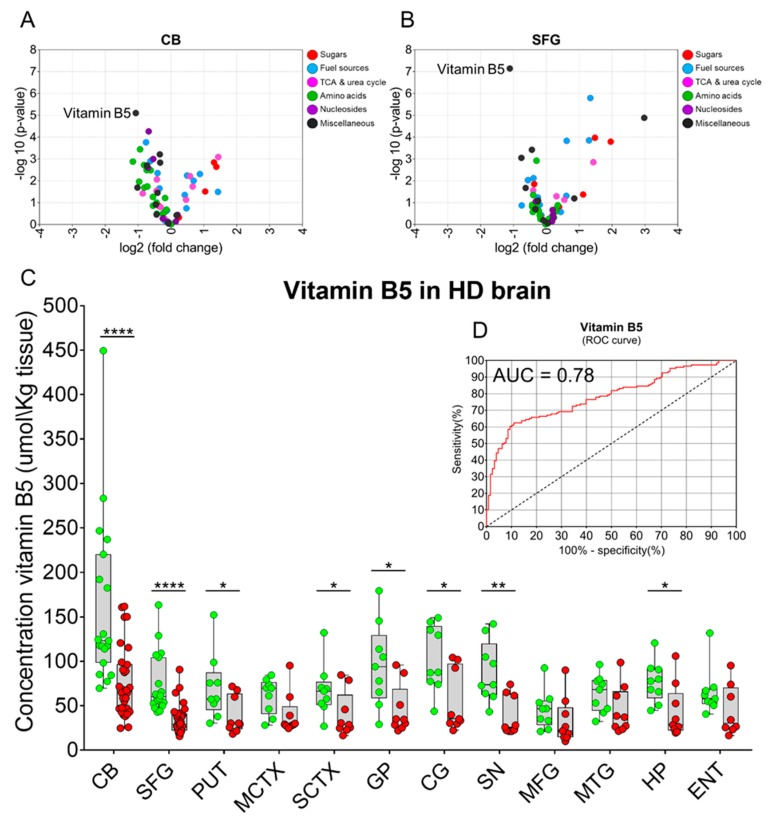
Evidence that vitamin B5 levels are decreased in multiple brain regions in HD. (**A**,**B**) Volcano plots showing individual metabolites and metabolite-groups dysregulated in HD brains in CB and SFG respectively. Vitamin B5 (D-pantothenic acid) was the most significantly altered metabolite identified in both brain regions. (**C**) Distributions of individual vitamin B5 concentrations (µmol/kg tissue) overlaying aggregated data (boxplots) in each of 12 brain regions of controls (green circles) and HD cases (red circles). Vitamin B5 concentrations are significantly reduced in CB, SFG, PUT, SCTX, GP, CG, SN, and HP (two-tailed *t*-tests for each region). (**D**) Receiver operating characteristic (ROC) curve for stratification of HD, based on vitamin B5 measurements was calculated using vitamin B5 concentrations extrapolated from a reference calibration curve (*n* = 278 brain samples). The AUC value was 0.78 with *p* < 0.0001. Here, the study group comprised all HD cases (*n* = 31, grades 0–4; red circles), and controls (*n* = 24; green circles); boxplots are means ± 95% CI. Abbreviations: *, *p* < 0.05; ** <0.005, ***, <0.001; ****, <0.0001; MCTX, motor cortex; MFG, medial frontal gyrus; MTG, medial temporal gyrus; ENT, entorhinal cortex.

Of the 63 small molecules quantified in CB samples, 40 had significantly changed levels in HD compared to control brains (*p* < 0.05 FDR-corr; Supplementary Table S2; Figure 4A). These alterations covered different classes of metabolites including sugars, fuel sources, TCA/urea-cycle-related intermediates, amino acids, nucleosides, and others. In SFG, 16 metabolites were found to be significantly altered in HD although nucleosides were not among the classes of small molecules altered in this brain region (*p* < 0.05 FDR-corr; Supplementary Table S2; Figure 4B). Of all the metabolites quantified in this study, vitamin B5 (pantothenic acid) showed the most marked change in HD in both brain regions examined (CB: *p* = 7.96 × 10^−6^ FDR-corr, SFG: *p* = 7.18 × 10^−8^ FDR-corr; Supplementary Table S2; Figure 4A,B). Vitamin B5 is located at the core of the CoA structure and hence plays a central role in CoA metabolism, and in all those downstream pathways using CoA as an essential co-factor [26]. To quantify abundance of vitamin B5 in brain samples, a calibration curve was generated by spiking known amounts of synthetic pantothenic acid into brain-derived background matrices, and this curve was then used to determine by interpolation the regional concentrations of this metabolite in tissue from control and HD-brain (R^2^ = 0.9951; Supplementary Figure S1). In addition to CB and SFG, we extended the quantification of vitamin B5 levels by this targeted assay system to other brain regions by measuring, retrospectively, the amounts of pantothenic acid in 10 additional brain regions [9]. Vitamin B5 concentrations in HD cases were significantly lower than in controls in 8 out of the 12 regions examined (CB, *p* = 6.2 × 10^−5^; SFG, *p* = 2.0 × 10^−6^; putamen (PUT), *p* = 0.041; sensory cortex (SCTX), *p* = 0.046; globus pallidus (GP), *p* = 0.013; cingulate gyrus (CG), *p* = 0.011; substantia nigra (SN), *p* = 0.002; hippocampus (HP), *p* = 0.017); the SN, a brain area belonging to the basal ganglia circuit, showed the greatest vitamin B5 decrease of all tissues examined (Figure 4A; Table 2). Overall, vitamin B5 concentration was significantly reduced to ~55% overall in the HD brain when compared to controls (*p* = 3.83 × 10^−12^ Table 2). Interestingly, decreased vitamin B5 was observed in HD mutation-carriers in brain areas commonly associated with minimal cell loss, namely CB, SFG, CG, and HP, indicating that the decline of vitamin B5 in the brain of HD cases is not just a result of generalized neurodegeneration. Furthermore, when comparing controls with HD cases with lesser degrees of brain pathology (Vonsattel grades 0/1/2), vitamin B5 concentrations were still significantly reduced, further supporting the hypothesis that cell loss may not play a major role in the vitamin B5 depletion observed in HD (Supplementary Figure S3). 

We also investigated whether the number of *HTT* CAG repeats had any effect on the regional levels of vitamin B5 in controls and HD brains by Spearman’s correlation analysis. Here, the correlations between the concentration of vitamin B5 and the *HTT* CAG lengths were considered significant if ρ-values were >0.8 (or <−0.8), with corresponding *p*-values of <0.01. *HTT* CAG size was not significantly related to vitamin B5 levels in controls or cases in any brain region examined (Supplementary Table S3); nor were vitamin B5 concentrations in any brain region significantly related to the Vonsattel grade, consistent with the view that the magnitude of cell loss or overall neurodegeneration does not contribute to the altered vitamin B5 levels observed in HD cases (Supplementary Table S3). Furthermore, age, PMD, or brain-weight not significantly related to regional brain vitamin B5 concentrations (Supplementary Table S3). Moreover, our qualitative observations indicate that neither individual cause of deaths (CODs) nor acute vs. chronic modes of death exerted a significant influence on regional vitamin B5 abundance in human brain in this study population (Supplementary Figure S4A,B). 

While the association of pantothenic acid deficiency with CNS diseases is largely unexplored, it has been shown that the impairment of CoA biosynthesis by non-utilisation of vitamin B5, is pathogenic in a sub-group of a larger family of neurodegenerative disorders called NBIA (neurodegeneration with brain iron accumulation), where an abnormal accumulation of this transition metal is observed in the basal ganglia [29]. To investigate if Fe and vitamin B5 are co-regulated in different brain regions in HD, we performed an exploratory correlation analysis using datasets generated by ICP-MS (inductively-coupled plasma MS). Overall, we observed an equal number of strong correlations between the HD and control groups, consistent with the absence of significant relationships between Fe levels and vitamin B5 concentrations, either in controls or those with HD (Supplementary Figure S5). 

## 3. Discussion

The motivation for this study was as follows. Age-related dementia has now outstripped heart disease as the leading cause of death in the United Kingdom and the rest of the developed world, according to the Office for National Statistics (https://www.ons.gov.uk). HD is one of seven major diseases responsible for age-related dementia, the others being AD, vascular dementia, Parkinson’s disease, Lewy body disease, frontotemporal lobar degeneration, and amyotrophic lateral sclerosis/motor neuron disease. Although each of these age-related dementias has disease-specific characteristics, there can also be considerable overlap between some cases. Currently, there are no disease-modifying therapies for any of these diseases, which cumulatively exert a huge toll on society [30]. The lack of disease-modifying therapies has suggested that new ways of looking at the pathogenesis of dementia are required [31]. 

Defects in the regulation of some low molecular-weight metabolites can impair their metabolism in brain and lead to deficiency states which, if sufficiently severe, may cause or resemble dementia or psychiatric disorders. Examples include deficiency of water soluble (B-group) vitamins including: thiamine (vitamin B1) [32]; niacin (vitamin B3) [33]; vitamin B6; folate (vitamin B9); [34] and cyanocobalamin (vitamin B12) [35]; and of the essential lipids [36]: linoleic acid [37]; and alpha-linolenic acid [38]. Therefore, the set of metabolites in the brain, termed the ‘brain metabolome’, is of interest as a target for further investigation in relation to the pathogenesis of dementia. Deficiency of the B-vitamins or essential fatty acids can cause widespread defects in brain function that are (usually) reversible by specific replacement therapies [26]. However, treatment of common age-related dementias, such as that caused by AD, with preparations containing B-vitamins (folate and vitamin B12) has proven ineffectual [39]. 

Considering these observations, we determined to undertake a systematic analysis of the low molecular-weight metabolome in the different age-related dementias, through a series of GC-MS-based case-control studies of post-mortem brain tissue. We have therefore developed a sensitive and specific methodology and optimized it for use in human brain tissue, to enable us to systematically identify and quantitate the accessible low-molecular-weight aspects of the brain metabolome [9,10,19,20]. 

We describe here the approaches whereby our GC-MS metabolomics platform was effectively optimised, and then used to profile metabolite abundances in human brain tissue that had been subject to long-term storage. We designed this study to assess key aspects of the quality of measurements obtained using the platform, namely intra-assay reproducibility, stability of metabolites during GC-processing time, platform stability over a year’s storage, and the durability of metabolites in brain specimens stored at −80 °C. Most intra-assay measurements had coefficients of variation (CVs) that were <15%, independent of the region used to generate QC technical replicates. This property is consistent with FDA guidance concerning acceptable standards for intra-assay precision indicated for bioanalytical method validation [40]. 

We also measured the stability-over-analysis-time and determined that all the monitored metabolite-group had CVs of <20% during the typical residence period (run time) of ~43 h. Other workers have found several metabolites to be prone to degradation (or conversion to new breakdown products) during the stand-by time in the autosampler [41]; here, however, metabolites with the analyte-classes selected did not change significantly over an extended and continuous MS acquisition time, supporting the proper optimisation of our workflow. 

The robustness and reproducibility of GC-MS platforms are also important aspects to consider when validating a proper workflow for metabolite profiling [42]. To assess these, we measured the metabolite abundances of the same replicate samples one year apart while, in the meantime, the instrument had been routinely employed inter alia to repeatedly analyse the rest of the brain samples included in this study. The same set of metabolites was consistently measured across all samples; perhaps more importantly, high correlation values were obtained across replicates, indicative of excellent reproducibility. Hence, these data show that our workflow is remarkably stable over this long time-period, a quality that is highly desirable in longitudinal studies. Our findings are consistent with those of others in diverse applications in the metabolomics field, and for other omics technologies [42,43]. 

The storage interval is another important variable that could potentially influence the correct measurement of analytes in tissues, and detailed studies concerning the effects of prolonged storage on brain samples are limited to date [44,45,46,47]. We therefore examined the effects of this component by measuring the metabolite levels in replicate tissue-samples at the beginning of our study and after one year of cryopreservation at −80 °C. As storage had no significant effects on the measured levels of analytes, we conclude that our metabolite profiling platform might be indicated to extend new and ongoing studies based on human brain tissue [48,49]. For instance, to our knowledge we were the first to apply an untargeted GC-MS-based global metabolomic approach to provide a detailed regional map of the metabolite alterations occurring in HD human brain [9]. 

While human brain analyses are still complicated by intrinsic elements which are difficult to control, for example post-mortem delay [50,51], new approaches are now under investigation to overcome these limitations [52,53]. 

The unsupervised cluster analyses and statistical modelling performed on data from these CB and SFG analyses indicate that profound metabolic alterations exist even in brain regions where cell loss in HD is not prominent. Of note, clustering of the metabolites, fructose, glucose-6-phosphate, urea, sorbitol, and glucose was evidently present in these data, replicating and extending our prior findings [9,19]. This concept is also supported by several recent in-vivo and ex-vivo studies [54,55]. Additional indications of dysregulated metabolism in moderately affected brain regions are provided by evidence of altered galactose, beta-alanine, and TCA-cycle regulation in HD mutation-carriers early in development of their disease, consistent with deficient energy metabolism in HD [56,57]. In the current study, we observed a marked decrease in vitamin B5, to an overall average of ~55% of corresponding control values, which could well lead to impairment of CoA biosynthesis in HD brain. Since CoA plays central roles in many important cellular processes including for example, the TCA cycle, pathways of fatty acid, porphyrin and polyamine metabolism, biosynthesis of neurotransmitters such as acetylcholine, and the regulation of amino acid, protein, RNA, and histone metabolism, it is possible that alterations in any of the steps in the CoA biosynthetic pathway might influence the proper functioning of such dependent processes. Interestingly, a group of disorders associated with the CoA biosynthetic pathway have the CNS as their primary target, for example, pantothenate kinase-associated neurodegeneration (PKAN), caused by pantothenate kinase 2 (PANK2) mutations, and HARP syndrome [25,58]; these are often characterised, like HD, by cognitive impairment, movement disorders, psychiatric features, and major brain-focussed neurodegeneration [59]. We note evidence that expression and/or activity of PANK1 may be a determining factor in the physiological regulation of the intracellular CoA concentration. Basal- ganglia iron levels are significantly increased in patients with HD [60], and a PKAN mutation is accompanied by prominent iron deposition in affected brain regions [61]. Here, we found brain levels of vitamin B5 and iron to be uncorrelated across all regions examined, indicating that elevated iron may not contribute to lowered vitamin B5 levels; these data also suggest that lowered vitamin B5 in HD may not be driven by altered PANK2 activity. Whether, and if so, how *HTT* mutations might lead to or cause vitamin B5 deficiency is currently unknown. Here, our correlational analysis of data from 296 brain-tissue samples found no relationship between CAG repeat number and vitamin B5 levels in cases or controls. Further studies, including measurements of tissue levels of CoA and acetyl-CoA by published methods [62], and of the five linked enzymes in the CoA biosynthetic pathway [28], are required to investigate the potential role of altered regulation of this pathway in HD. 

The presence of severe elevations in levels of glucose, sorbitol, fructose, glucose-6-phosphate, and urea in the putamen of HD brain, as reported in our previous publications [9,19,20], and additionally supported here by results of unsupervised cluster analysis, point to increased flux in the polyol pathway [9], coupled with defective utilization of glucose via glycolysis, wherein there is a presumptive block distal to the hexokinase-catalyzed glucose-phosphorylation step. 

This pattern particularly affects high-impact brain regions, for example, the striatum, and closely resembles a defect-pattern we recently discovered in AD [10]. Under physiological circumstances, most glucose entering the glycolytic pathway in brain is destined to enter the TCA cycle via pyruvate dehydrogenase-catalysed formation of acetyl-CoA [27], which is in turn derived from its obligatory precursor, vitamin B5 [28]. A defect in this pathway, evoked by defective vitamin B5 homeostasis leading to impaired CoA formation, provides a potential mechanism underlying these observations. This hypothesis will need to be tested by direct measurement of pathway components in future case-control studies in animal models and human brain tissues. 

Here, urea is clustered with polyol-pathway intermediates and glucose-6-phosphate, pointing to potentially interlinked defects in their related metabolic pathways. It is not known whether brain tissue contains a complete, functioning urea cycle, and the origin of the elevated brain urea in HD, and indeed in AD, remains uncertain. However, several enzymes relevant to urea biosynthesis and metabolism, including carbamoyl phosphate synthetase, N-acetylglutamate synthase, carbonic anhydrase, and glutamate dehydrogenase are expressed therein [19,20]. Moreover, there are numerous molecular links between the TCA cycle and urea metabolism, and substantive evidence that co-operation of these pathways is dependent upon acetyl-CoA as a key metabolite common to both [63,64]. 

Here we identify substantive loss of vitamin B5 from the basal ganglia and regions functionally linked through basal ganglia-thalamocortical circuits, including frontal and sensory cortices, globus pallidus, putamen, substantia nigra, and cingulate gyrus [65]. Interestingly, the basal ganglia, which are primarily affected by severe cell loss in HD, begin to degenerate before clinical symptoms are clearly manifest during disease progression [66,67]. Brain levels of vitamin B5 are typically ~50-fold higher than those in plasma [62]; these stores likely reflect high demand for CoA to support cell metabolism, for example in the synthesis of acetylcholine, and may well indicate binding and sequestration in intracellular structures, to lower a hypothetical ‘free’ fraction to facilitate uptake through the membrane-bound SLC5A6 transporter. 

Our results point to a possible defect in the mechanism of cerebral uptake and/or storage of vitamin B5, consistent with its lowered concentrations in affected regions of HD brain. Whereas certain genetically-transmitted disorders causing defects in the CoA synthetic pathway occur in humans, the effects of chronic vitamin B5 depletion on the brain are poorly understood [68,69]. The human sodium-dependent multivitamin transporter (hSMVT), encoded by the *SLC5A6* gene, mediates uptake of pantothenate along with biotin, lipoate, and iodide [70]. Human SMVT is the main transporter of pantothenate through the blood-brain barrier [71,72], and loss-of-function mutation in *SLCA6* in humans causes brain dysfunction, amongst other phenotypic effects [73]. These observations, taken together with the finding of deficient vitamin B5 in HD brain, point to hSMVT as a target for further investigation in relation to the pathogenesis of HD. 

Acetylcholine is a major neurotransmitter in brain that is localized in cholinergic neurons. It is formed by acetyl-group transfer from acetyl-CoA to choline, catalysed by choline O-acetyl transferase (ChAT; EC 2.3.1.6) [74]. Most acetylcholine in mammalian brain is bound [75] and sequestered by action of the vesicular acetylcholine transporter, SLC18A3 in secretory organelles in nerve terminals [76]. The subcellular localization of vitamin B5 in brain is uncertain at present. We hypothesize that since it is an obligatory precursor of acetyl-CoA and hence acetylcholine, for which there is a high demand in brain, then its stores might also be localized in cholinergic neurons, at least in part. Co-localization studies will be required to test this hypothesis. 

Acetyl-CoA has other crucial regulatory functions that may be relevant to interpretation of our current findings. For example, it acts as a metabolic regulator at the interface between carbohydrate, fat, and protein catabolism that is rapidly depleted upon starvation [77]. Acetyl-CoA depletion in turn, causes a reduction in the overall acetylation of cytoplasmic proteins, along with induction of autophagy, a homeostatic process of self-digestion, and cytosolic acetyl-CoA may act as a central metabolic regulator of autophagy [77]. Acetyl-CoA is a main acetyl-donor for the acetylases that catalyse histone acetylation, and thereby contributes to gene regulation. It has been thought that glucose-derived carbon is the main source of acetyl-CoA used for histone acetylation. However, there is recent evidence that lipids can provide a major carbon source for histone acetylation [78]. Fatty acid oxidation was found to increase global histone acetylation, and lipids were shown to provide up to 90% of acetyl-carbon for histone acetylation, which is mainly provided by octanoate; it was also shown that lipid-derived acetyl-CoA enhances expression of lipid-metabolizing enzymes [79]. These findings extend current understanding of the roles of acetyl-CoA in nutrient sensing and metabolic regulation. 

This study has limitations. First, the study population of 30 HD cases and 19 controls, comprised 49 subjects in all; therefore, it requires further replication in independent groups of cases and controls to avoid inaccuracies in interpretation caused by the relatively small sample size [80,81]. However, lowered vitamin B5 levels were present in multiple, functionally distinct brain regions including those markedly different in levels of damage, so neurodegeneration per se is unlikely to provide enough of an explanation for these lowered tissue concentrations. The *p*-value associated with the main effect in aggregate data from all regions is 3.8 × 10^−12^, providing evidence that substantive multi-regional lowering of vitamin B5 occurs in HD brain. This study cannot determine whether the identified metabolic perturbations, in particular vitamin B5 deficiency, might themselves cause tissue damage, or whether they occur as downstream epiphenomena in the pathogenic process, and are not involved in the mechanism of neurodegeneration. However, substantial available genetic evidence points to neurodegeneration caused by mutations in a central enzyme of the synthetic pathway that leads from vitamin B5 to CoA, and also in the vitamin B5 transporter itself, providing robust evidence for linkages between defects in vitamin B5 regulation and neuropathology [82]. Additionally, the current findings do not explain how *HTT* mutations might cause vitamin B5 deficiency or provide any mechanistic insights as to how the two processes might be linked. While the examination of HD brain samples with moderate cell loss suggests that impaired vitamin B5 regulation might occur early in the HD process, studies in greater numbers of cases of low pathological grade HD will be needed before these results are confirmed. 

In conclusion, we have developed and validated a robust and reproducible method for identifying and measuring major metabolites present in the low-molecular-weight fraction of the metabolome in human brain, and have used it here to analyse a case-control study that aimed to replicate and extend our earlier studies of metabolic perturbations in HD as a window on the pathogenesis of chronic age-related dementia. Through these studies, we have identified a novel metabolic perturbation, vitamin B5 deficiency, which has the potential to cause neurodegeneration through impairments in the regulation of acetyl-CoA, for which it is the obligatory precursor. This finding in the human brain is reported here, for the first time to our knowledge. We think this finding is important, because it raises the possibility that part of the pathogenic process in *HTT*-mediated dementia could be evoked through defective vitamin B5 metabolism, leading in turn to impaired acetyl-CoA metabolism, and downstream tissue damage elicited through the multitudinous pathways it regulates. If vitamin B5 deficiency is indeed part of the pathogenesis in patients with *HTT* mutations, then its replacement might be a novel intervention with the potential to halt or suppress neurodegeneration itself, analogous to the effects of replacement of other B-vitamins in scenarios where a specific deficiency disorder is causative of neurodegeneration, for example vitamin B12 deficiency. We think it is now timely to begin consideration of evidentiary paths that will ultimately lead to trials of vitamin B5 therapy in disease caused by *HTT* mutation. Such intervention could be initiated before *HTT* mutation becomes manifest as Huntington’s disease. 

## 4. Materials and Methods

### 4.1. Acquisition of Human Brains

We obtained tissue samples from 30 HD cases and 19 controls from the New Zealand Neurological Foundation Human Brain Bank, in the Centre for Brain Research, Faculty of Medical and Health Sciences, University of Auckland, Auckland, New Zealand. All families gave informed consent and the University of Auckland Human Participants Ethics Committee approved all procedures employed. 

### 4.2. Human Brain Tissue

The human brain dissection method applied here was previously described [9]. In brief, each brain region was identified and accurately micro-dissected by trained neuroanatomists. We collected tissue from two brain regions for the main metabolomic studies: cerebellum, CB; and superior frontal gyrus, SFG. We dissected tissue samples of 50 ± 5 mg from each region from each subject, and these were stored at −80 °C until analysis. 

### 4.3. Tissue Extraction for GC-MS

We used a previously described method [9,19] by which aliquots of 50 ± 5 mg tissue were extracted in 0.8 mL of 50:50 (*v*/*v*) methanol:chloroform, to which a solution of isotopically-labelled internal standards (citric acid-d4, 13C6-D-fructose, L-tryptophan-d5, L-alanine-d7, stearic acid-d35, benzoic acid-d5, and leucine-d10) in methanol was added to final concentrations of 0.016 mg/mL of each internal standard in the extraction solvent. We blended tissue samples (TissueLyser bead homogeniser; Qiagen, UK) for 10 min at 25 Hz with a 3 mm tungsten carbide bead and did phase separation by addition of 0.4 mL water followed by vortex-mixing (10–15 s) and centrifugation (2400 *g*, 5 min), with subsequent removal of the chloroform layer. From the methanol:water supernatant, 200 μL aliquots were transferred to pre-labelled tubes and quality control replicates were prepared by pooling equal amounts of extracts from each sample, with subsequent drying of aliquots (Speedvac centrifugal concentrator; Thermo-Fisher, Waltham, MA, USA) and storage at 4 °C until derivatization for GC-MS analysis. 

### 4.4. GC-MS and Data Analysis

We have previously detailed the method used for GC-MS metabolomic analysis [82,83]. Briefly, methoximation and trimethylsilylation were used to generate a profile of polar small-molecule metabolites such as those corresponding to amino acids, simple organic acids, and monosaccharides, this approach is frequently used to generate comparative data using case-control study designs [84]. Data were processed with ChromaTOF 4.5 (LECO) and MS outputs matched against two publicly available spectral libraries: the Golm Metabolome Database (Max Planck Institute of Molecular Plant Physiology, Potsdam-Golm, Germany); and the NIST Mass Spectral Reference Library (NIST08/2008; National Institute of Standards and Technology/Environmental Protection Agency/National Institutes of Health Spectral 262 Library; NIST, Gaithersburg, MD, USA). We monitored run-to-run, batch-to-batch, and in-time chromatographic variation by examination of retention-time variability of internal reference standards. To determine an unambiguous metabolite identification of the spectra matched by our software, two independent investigators manually verified mass spectra/expected retention times and peak-shape integration. 

### 4.5. Tissue Extraction for Inductively Coupled Plasma Mass Spectrometry (ICP-MS)

We applied a previously optimised ICP-MS-based method to determine metal concentrations normalized on a per-dry-weight basis in tissue from nine anatomically-verified regions: PUT, MCTX, SCTX, GP, SN, MFG, MTG, HP, and ENT. Samples of 50 ± 5 mg wet-weight were dehydrated to constant weight in a centrifugal concentrator (Speedvac) and wet-to-dry-weight ratios determined. We digested dried tissue in 2 mL microcentrifuge tubes (Eppendorf) using concentrated nitric acid (A509 Trace Metal Grade; Fisher, Loughborough, UK) with added 5% (*v*/*v*) Agilent Internal Standard mixture (5183-4681; Agilent Technologies, Cheadle, UK). We punctured tube lids to prevent pressure build-up, added standard-containing nitric acid, and then progressively heated tubes from room temperature to 60 °C for 30 min in a dry-heating block; thereafter, we increased the set temperature to 100 °C for a further 210 min. Once tissue digestion was complete, we allowed tubes to cool overnight. To provide a true “digestion” blank, tissue-free tubes containing standard-acid solution were included in each batch, and appropriate dilutions of internally-standardized acid provided rinse and calibration solutions, at 2% (*v*/*v*) final nitric acid concentration. We produced calibration solutions by appropriate dilutions of Environmental Calibration Standard (Agilent 5183-4688). 

### 4.6. ICP-MS and Data Analysis

Metal concentrations were determined using an Agilent 7700x ICP-MS spectrometer equipped with a MicroMist nebulizer (Glass Expansion, Melbourne, Australia) and a Scott double-pass spray chamber, with nickel sample and skimmer cones. We introduced samples using an Agilent Integrated autosampler (I-AS) with helium (He) as the collision gas and applied a multi-element method including all those present in the calibration solution, as previously described [85], and used scandium as the internal standard for all elements. We applied two collision-cell gas modes: all elements were analysed in helium mode (5.0 mL·min^−1^ He). Mode selection followed Agilent’s recommendations to minimize interference for measured elements by, e.g., isobaric cluster ions. Integration times were 0.01 s for Fe. For each analytical batch, we performed multi-element calibration using serial dilutions of the calibration standard. We used an intermediate concentration from this calibration series as a periodic QC sample throughout each analytical batch. Instrument and digestion blanks were also interspersed through each set of randomized samples. We determined the detection limit for Fe by comparison of calibration samples and blanks, and any samples below this level were eliminated prior to reporting. 

### 4.7. Vitamin B5 Concentration Measured by Targeted GC-MS Assay

Chromatographic retention-time data for vitamin B5 were available from our in-house library of reference standards. To constitute a definitive molecular identification, the correct matching of both mass spectra/expected retention time and peak-shape integration was manually verified by two independent investigators (Supplementary Figure S1A). 

For vitamin B5, we built a calibration curve using known quantities of authentic synthetic standards to assess the compound stability on our analytical platform (Sigma-Aldrich; Supplementary Figure S1B). To allow quantitative estimation of vitamin B5 in human brain matrices, an additional calibration curve was created by adding pure pantothenic acid standards into sample extracts (Supplementary Figure S1C). Vitamin B5 levels were calculated and data have been presented here as µmol/kg of fresh brain tissue. 

### 4.8. Data Analysis and Statistics

In the current study, in-house bioinformatic algorithms implemented in R (version 3.5.1) and Python (version 3.6) were used to perform data mining, data visualisation and statistics, and to explore the experimental results. Analytical platform reproducibility was evaluated by calculating the % coefficient-of-variation (%CV) of each metabolite in QC replicates. Here %CV < 20% was chosen as the proper cut-off to determine good reproducibility. Platform stability was estimated by calculating correlation coefficients between the relative abundance of each metabolite (log10transformed) in processed replicates of QC samples, and brain samples after long-term storage, respectively. Pearson’s correlation coefficient (r) was used to provide surrogate measurements of the reproducibility and correlations were considered significant only if both r- and corresponding *p*-values were at least >0.95 and <0.001, respectively. Multivariate PCA and OPLS-DA were performed using R (MetaboAnalystR; version 1.0.1) to assess data quality and identify global chemical differences across multiple brain regions in HD cases and controls. To assess the validity of the OPLS-DA, goodness-of-fit values of the model were calculated and then compared with the goodness-of-fit of 100 Y-permutated models. The VIP score, a measure of a variable’s importance in the OPLS-DA model, was then calculated and its values used to pinpoint the major metabolites contributing to the differentiation observed in the OLPS-DA model between HD and non-demented controls. A Mann–Whitney U test (*p* < 0.05) was applied to test significance in the model. Multiple 2-tailed *t*-tests were applied to determine if relevant metabolite-abundance differences were observed, and to ascertain the statistical significance of our observations, we considered our entire datasets for multiple-comparison analyses by applying a 10% false-discovery rate correction [86]. We calculated fold-changes in metabolite abundances as the ratios of the means of each case-control group and have presented them here as HD-group/control-group ratios. Hierarchical clustering (HC) analyses were constructed using in-house scripts implemented in R packages (ggplot2 v2.2.1; pheatmap v1.0.8; heatmap.plus v1.3; lattice v0.20-0.35; magrittr v1.5), and the Euclidean distance method was applied to generated clustering heat-maps. Non-parametric Mann–Whitney U-tests and receiver-operating characteristic (ROC) curves (GraphPad v6.04 Prism; La Jolla, CA) were used to assess the ability of vitamin B5 to discriminate between HD and healthy controls. The area-under-the-curve value (AUC) was calculated by numerical integration of the curve. The significance of the corresponding *p*-value was set at 0.001, and the AUC cut-point at 0.75. In each brain region, the potential effect of the *HTT* allele CAG repeat size on vitamin B5 abundance was investigated, by generating Spearman’s correlations between the number of CAG triplets and the concentration of vitamin B5, using implementations of statistical and data mining scripts available in Python modules (Pandas v0.23.3; NumPy v1.15.0; Matplotlib v2.2.2; SciPy v1.1.0; and Seaborn v0.9.0). A correlation was considered significant only if both ρ- and *p*-values were at least >0.8 (or <−0.8) and <0.01, respectively. Similar criteria were used to investigate how brain pathology grade (Vonsattel), age, PMD and brain-weight might relate to the regional vitamin B5 concentration in human brain. For ICP-MS analyses, datasets were exported to Microsoft Excel worksheets and individual values of each sample normalized by the corresponding sample dry-weight. Weight-adjusted datasets were then log10-transformed for statistical analysis. Means (±95% CI) of the log10-transformed data were calculated, and the significance of between-group differences was examined by unpaired Welch’s *t*-tests to allow for unequal variances and sample sizes. Means (±95% CI) were back-transformed to reflect the actual elemental concentrations. Statistical calculations and data visualisation were performed using GraphPad v6.04 (Prism; La Jolla, CA, USA). Correlation differences were statistically assessed by Fisher r-to-z transformation method and *p*-values < 0.05 (two-tailed) were considered significant. 

## Figures and Tables

**Table 1:** Table showing study-group characteristics.

Variable	Controls	HD
Number	19	30
Age (y; ±95% CI)	61.1 (54.1–68.0)	58.9 (54.8–63.1)
PMD (h; ±95% CI)	17.7 (14.8–20.7)	14.7 (11.8–17.5)
Brain-weight (g; ±95% CI)	1327.4 (1283.5–1371.3)	1091.4 (1032.2–1150.7) *

Data are means (±95% CI); * *p*-value < 0.0001. PMD, post-mortem delay.

**Table 2:** Vitamin B5 concentration levels in controls and HD cases. Table illustrating the mean (±95% CI) values of vitamin B5 concentration for each brain area analysed in controls and HD gene-carriers. The overall mean of vitamin B5 concentration across all the brain areas is also reported in the table. Concentration values have been expressed as µmol/kg brain tissue. Multiple two-tailed *t*-tests were used and *p*-values < 0.05 were considered significant. Vitamin B5 fold-changes between HD versus controls are included in the table.

Brain Region	Vitamin B5 Control (µmol/kg Tissue)	Vitamin B5 HD (µmol/kg Tissue)	*p*-Values	Fold-Change (HD vs. Control)
CB	161.7 (116.4–206.9)	76.6 (61.7–91.4)	**6.2 × 10^−5^**	0.5
SFG	73.6 (57.6–89.6)	34.7 (28.4–41.1)	**2.0 × 10^−6^**	0.5
PUT	71.0 (42.6–99.3)	39.5 (23.4–55.5)	**0.041**	0.6
MCTX	60.9 (45.6–76.3)	40.2 (22.3–58.1)	0.060	0.7
SCTX	67.5 (45.2–89.9)	39.8 (20.5–59.1)	**0.046**	0.6
GP	95.7 (59.7–131.7)	45.5 (24.4–66.5)	**0.013**	0.5
CG	103.2 (74.6–131.8)	54.4 (28.0–80.8)	**0.011**	0.5
SN	88.1 (61.9–114.3)	38.1 (21.2–55.0)	**0.002**	0.4
MFG	45.6 (28.9–62.2)	32.4 (12.4–52.4)	0.262	0.7
MTG	63.5 (47.4–79.6)	45.1 (24.7–65.4)	0.121	0.7
HP	76.7 (58.9–94.5)	43.1 (20.2–66.0)	**0.017**	0.6
ENT	65.4 (45.1–85.8)	44.4 (20.6–68.3)	0.137	0.7
**Mean**	**86.8** (76.9–96.7)	**47.63** (43.1–53.4)	**3.831 × 10^−12^**	0.5

## Supplementary Materials

The following are available online at https://www.mdpi.com/2218-1989/9/6/113/s1.
