# Supplementary material for: Cerebral Vitamin B5 (D-Pantothenic Acid) Deficiency as a Potential Cause of Metabolic Perturbation and Neurodegeneration in Huntington’s Disease
Source: Metabolites. 2019 Jun 11;9(6):113. doi: 10.3390/metabo9060113 (PMC6630497; doi:10.3390/metabo9060113)
Supplement: Supplementary file 1 [file metabolites-09-00113-s001.pdf]

metabolites

Supplementary materials

Cerebral vitamin B5 (D-pantothenic acid) deficiency as a potential cause of metabolic perturbation and neurodegeneration in Huntington's disease

Stefano Patassini [1, 4, 6], Paul Begley [1], Jingshu Xu [1, 2], Stephanie J. Church [1], Nina Kureishy [1], Suzanne J. Reid [3, 4], Henry J. Waldvogel [3], Richard L. M. Faull [3], Russell G. Snell [3, 4], Richard D. Unwin [1] and Garth J. S. Cooper [1, 2, 4, 5*].

[1] Centre for Advanced Discovery and Experimental Therapeutics, Division of Cardiovascular Sciences, School of Medical Sciences, Faculty of Biology, Medicine & Health, The University of Manchester, Manchester, United Kingdom

[2] Manchester Cancer Research Centre Building, The University of Manchester, Manchester, United Kingdom

[3] Centre for Brain Research and Department of Anatomy and Medical Imaging, Faculty of Medical and Health Sciences, University of Auckland, Auckland, New Zealand

[4] School of Biological Sciences, Faculty of Science, University of Auckland, Auckland, New Zealand

[5] Maurice Wilkins Centre for Molecular Biodiscovery, University of Auckland, Auckland, New Zealand

[6] Present address: Owlstone Medical, Cambridge Science Park, Cambridge, United Kingdom

*Correspondence: garth.cooper@manchester.ac.uk

N	Status	Age	Gender	Cause of death	Grade	CAG repeats	PMD (h)	Brain Weight (g)
1	Control	42	M	Chest trauma	-	N/D	14	1403
2	Control	61	M	Ischaemic heart disease	-	17/19	7	1258
3	Control	72	F	Myocardial infarction	-	17/19	19	1264
4	Control	63	F	Aortic aneurysm	-	14/16	16	1324
5	Control	73	M	Ischaemic heart disease	-	17/23	13	1315
6	Control	89	M	Coronary atherosclerosis	-	17/19	19	1430
7	Control	66	M	Ischaemic heart disease	-	15/20	15	1360
8	Control	77	F	Ischaemic heart disease	-	N/D	13	1184
9	Control	81	M	Coronary atherosclerosis	-	15/18	7	1343
10	Control	43	F	Nitrogen poisoning	-	17/17	26	1318
11	Control	59	M	Aortic aneurysm	-	17/18	24.5	1490
12	Control	60	M	Ischaemic heart disease	-	10/17	17	1370
13	Control	48	M	Ischaemic heart disease	-	17/20	23	1470
14	Control	53	M	Ischaemic heart disease	-	N/D	16.5	1215
15	Control	78	F	Aortic aneurysm	-	18/19	20	1292
16	Control	56	M	Asphyxia	-	N/D	23	1358
17	Control	57	F	Carcinomatosis	-	N/D	32	1243
18	Control	41	M	Heart disease	-	N/D	16	1171
19	Control	41	M	Asphyxia	-	18/22	16	1412
20	HD	54	M	Pneumonia	2	20/39	6.5	1272
21	HD	67	F	Myocardial infarction	1	15/42	9	1139
22	HD	59	F	Pneumonia	4	23/47	7	787
23	HD	62	F	Pneumonia	3	17/45	11	826
24	HD	62	M	N/D	2	18/43	9	992
25	HD	83	M	Pneumonia	1	17/42	13	1168
26	HD	58	M	Pneumonia	2	28/44	14	1497
27	HD	51	M	Pneumonia	2	10/46	15	1200
28	HD	65	M	Renal failure	2	17/43	14	1224
29	HD	63	M	Pulmonary embolism	3	22/43	16	1226
30	HD	45	F	Choking	2	24/43	15	1014
31	HD	64	M	Pulmonary embolism	3	27/42	20.5	1252
32	HD	53	F	Pneumonia	2	21/47	12	961
33	HD	45	M	Pneumonia	4	20/49	18	950
34	HD	72	M	Pneumonia	1	17/41	5	1190
35	HD	53	F	Pneumonia	4	17/53	9	1010
36	HD	56	M	Pneumonia	2	16/46	16	1053
37	HD	44	M	Pneumonia	4	20/51	29	1147
38	HD	48	M	Pneumonia	3	20/45	18	1010
39	HD	64	M	Pneumonia	3	18/44	19	1250
40	HD	63	F	N/D	3	23/44	5.5	955
41	HD	51	M	Dehydration	3	17/48	15.5	1007
42	HD	71	M	Pneumonia	2	19/42	16	1259
43	HD	50	F	Pneumonia	2	16/46	20	1120
44	HD	91	F	Dehydration	2	15/40	18	869
45	HD	65	M	Pneumonia	2	18/46	6	900
46	HD	57	F	Myocardial infarction	2	17/44	19	1085
47	HD	62	M	Pulmonary embolism	0	27/41	19	1180
48	HD	43	F	Renal failure	3	21/49	3.5	970
49	HD	47	M	Myocardial infarction	3	19/51	41	1230

Supplementary Table S1. Table illustrating clinical information and metadata for the cases used in this study. Abbreviation: *PMD*, post-mortem delay; *N/D*, not declared.

Group of metabolite	CB		SFG	
	Fold change	p-value	Fold change	p-value
**Glucose metabolites & pentoses**				
Fructose	2.5	0.0015	2.8	0.0001
Sorbitol	2.6	0.0023	2.2	0.0426
Glucose	1.2	0.4754	1.3	0.1602
Glucose-6-phosphate	2.1	0.0312	3.9	0.0002
Ribose-5-phosphate	0.8	0.1371	0.8	0.0141
**Alternative fuel source**				
β-Hydroxybutyric acid	1.4	0.1802	1.4	0.2703
Glycerol	0.8	0.0045	0.8	0.0577
Threitol	1.4	0.0057	1.5	0.0001
Glycerol-3-phosphate	1.8	0.0049	2.5	0.0001
Glycerol-2-phosphate	1.3	0.0447	2.5	<0.0001
Scyllo-inositol	0.6	0.0002	0.7	0.0093
Myo-inositol	0.9	0.2286	0.9	0.3818
N-acetylglucosamine	0.9	0.6699	1.0	0.8770
Ribitol	0.6	0.0013	0.8	0.0075
Arabitol	0.8	0.0224	0.9	0.1252
Mannitol	1.6	0.0099	1.5	0.0493
Lactic acid	1.0	0.9306	1.1	0.7432
Disaccharide	2.7	0.0322	0.6	0.1356
**TCA & Urea cycle and related**				
Fumaric acid	1.5	0.0061	1.2	0.0510
Citric acid	1.6	0.0181	1.1	0.3966
Malic acid	1.4	0.0733	0.9	0.4695
Urea	2.7	0.0008	2.7	0.0014
Ornithine	0.5	0.0377	0.9	0.5132
N-acetylglutamic acid	0.7	0.0088	0.8	0.0273
Creatinine	0.8	0.1590	1.0	0.9226
Pyruvic acid	0.9	0.5431	1.5	0.0743
Succinic acid	0.7	0.0290	1.0	0.8449
**Amino acids**				
Glycine	0.8	0.3254	0.9	0.3828
Leucine	0.7	0.1381	1.0	0.8292
Isoleucine	0.6	0.0019	0.7	0.1506
Serine	0.5	0.0004	0.8	0.0450
Threonine	0.7	0.0033	0.8	0.1175
Aspartic acid	0.6	0.0204	0.8	0.1207
Methionine	0.4	0.0013	0.8	0.2702
Pyroglutamic acid	1.0	0.9832	1.0	0.8469
Phenylalanine	0.7	0.0989	1.2	0.2461
Proline	0.7	0.0545	0.9	0.5670
N-acetylaspartic acid	0.9	0.0621	0.8	0.0012
Lysine	0.5	0.0110	1.0	0.9754
Tyrosine	0.6	0.0183	1.2	0.4759
Tryptophan	0.9	0.2758	1.2	0.1897
Glutamine	1.0	0.9610	1.1	0.5474
Alanine	0.9	0.2079	0.9	0.2331
Beta-alanine	1.0	0.9161	1.0	0.9270
Cysteine	0.7	0.3225	1.3	0.1305
Valine	0.6	0.0032	0.7	0.1250
**Nucleosides**				
Uracil	0.7	0.0010	0.8	0.0852
Hypoxanthine	0.6	0.0001	0.8	0.0874
Guanosine	0.8	0.5378	N/A	N/A
Xanthine	0.6	0.0025	1.2	0.3615
Inosine	0.9	0.7367	1.2	0.5014
Adenine	1.1	0.3650	1.1	0.2165
Adenosine	1.1	0.7265	N/A	N/A
**Miscellaneous**				
Ethanolamine	0.6	0.0020	0.6	0.0009
Phosphoric acid	0.8	0.0006	0.7	0.0004
GABA	0.8	0.0015	0.8	0.0814
4-hydroxybutyric acid	0.5	0.0206	1.0	0.8531
Vitamin B5	0.5	<0.0001	0.5	<0.0001
Glutaric acid	0.8	0.0359	0.9	0.6399
Ethylene glycol	0.7	0.3541	0.6	0.0215
Sugar phosphate	0.9	0.8948	7.9	<0.0001
Gluconic acid	0.7	0.1218	1.8	0.0643
Methyl-phosphate	1.1	0.3796	0.8	0.1991

Supplementary Table S2. Metabolites altered in abundance across the two brain regions analysed by GC-MS. Metabolites analysed in this study are listed and illustrated as separated metabolite-groups in the table. For each compound, fold-changes are reported as HD group/control group. After being tested by multiple comparison analysis (FDR-corrected), the metabolites significantly altered in abundance in HD were highlighted. In red are listed the compounds increased in HD and in blue those that are decreased.

Brain region	CAG size (Control)		CAG size (HD)		Vonsattel grade (only HD)		Age		PMD		Brain-weight	
	Spearman	p-value	Spearman	p-value	Spearman	p-value	Spearman	p-value	Spearman	p-value	Spearman	p-value
**CB**	0.12	0.7100	0.17	0.3700	0.33	0.0800	-0.02	0.9100	0.10	0.4900	0.41	<0.0050
**SFG**	-0.20	0.5100	-0.01	0.9600	0.20	0.3100	0.05	0.7300	0.18	0.2200	0.45	<0.0050
**PUT**	0.50	0.1800	0.14	0.7200	0.11	0.8000	0.29	0.2600	-0.30	0.2300	0.18	0.4700
**MCTX**	0.52	0.1600	-0.11	0.7900	-0.26	0.5000	0.25	0.3200	-0.20	0.4200	0.16	0.5200
**SCTX**	0.19	0.6300	-0.02	0.9800	-0.11	0.8000	0.08	0.7500	-0.30	0.2400	0.09	0.7200
**GP**	-0.15	0.7000	-0.28	0.4700	-0.34	0.3700	0.44	0.0700	-0.12	0.6400	0.23	0.3600
**CG**	0.51	0.1700	-0.15	0.7000	-0.24	0.5400	0.26	0.3000	-0.34	0.1800	0.29	0.2500
**SN**	0.10	0.8000	-0.24	0.5500	-0.24	0.5400	0.35	0.1600	-0.22	0.3900	0.41	0.1000
**MFG**	0.35	0.3600	0.06	0.8900	0.14	0.7200	0.08	0.7600	-0.31	0.2200	0.14	0.5900
**MTG**	0.23	0.5500	-0.06	0.8900	0.06	0.8800	0.18	0.4800	-0.39	0.1200	0.17	0.5100
**HP**	0.34	0.3700	-0.17	0.6700	0.00	>0.9999	0.35	0.1600	-0.54	0.0300	0.10	0.7100
**ENT**	0.20	0.6100	0.08	0.8600	0.00	>0.9999	0.12	0.6600	-0.28	0.2900	-0.08	0.7600

Supplementary Table S3. Vitamin B5 shows no correlation with multiple study-group characteristics. Table showing the Spearman's coefficients (ρ) and p-values obtained correlating vitamin B5 concentrations with different study-group characteristics. A correlation was considered significant only if ρ values >0.8 (or <-0.8) and p-values <0.01. The *HTT* CAG size of the longest allele was considered to assess potential correlations between vitamin B5 concentrations and *HTT* CAG repeats in controls and HD cases. For Vonsattel grade only HD cases were considered for correlation analyses. For age, PMD and brain-weight all the subjects included in the study were considered. No significant correlations of vitamin B5 with *HTT* CAG size, Vonsattel grade, age, PMD and brain-weight were observed in any of the brain regions analysed by GC-MS.

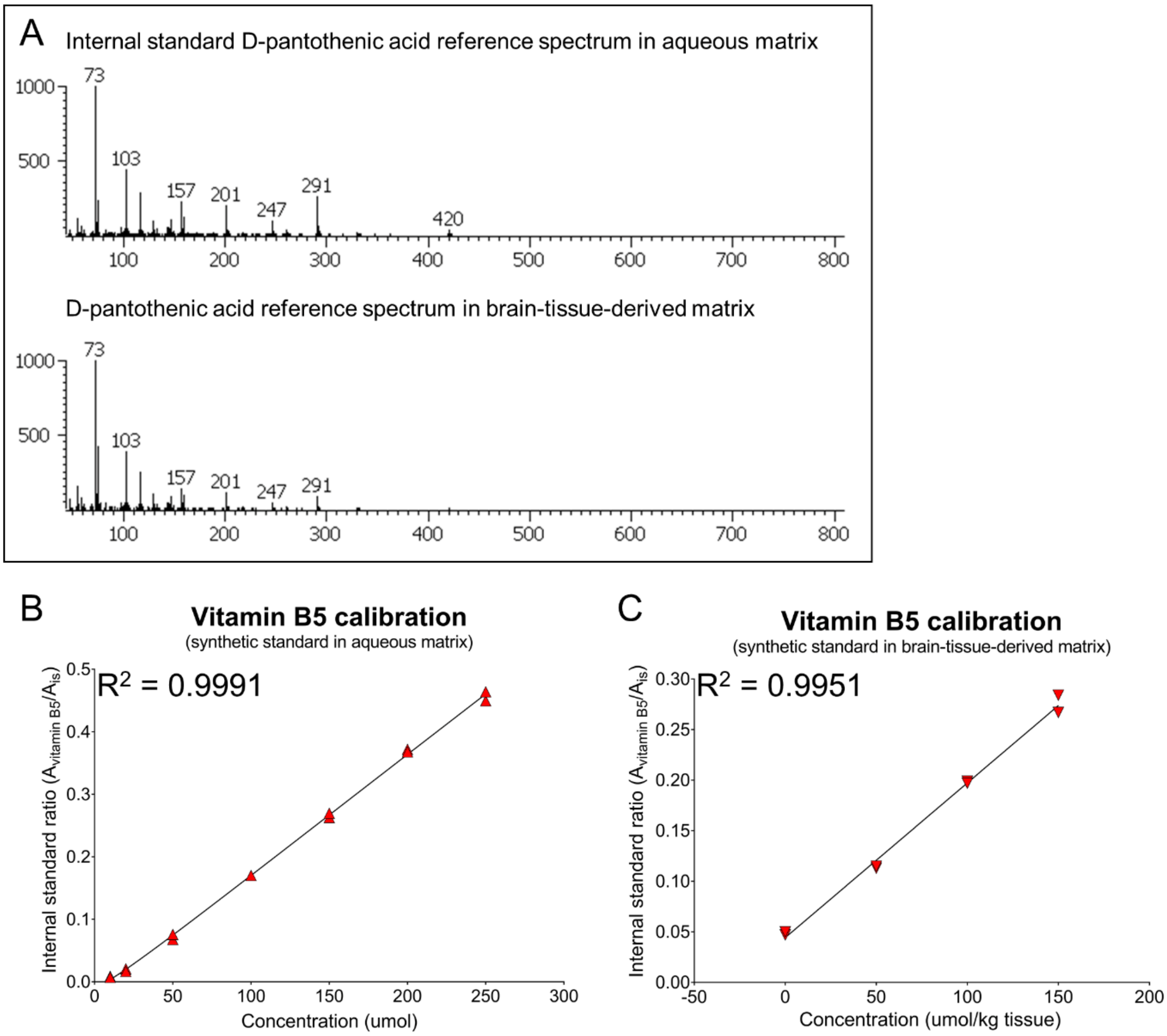

Supplementary Figure S1. Electron ionization (EI) spectra and calibration curves for vitamin B5 (D-pantothenic acid) derived from aqueous or human-brain-derived matrices. (**A**) Spectra showing EI fragmentation of analytical-grade D-pantothenic acid by GC-TOF derived from a solution in an aqueous matrix (top panel) or after addition to human-brain-extract pool (bottom panel). A library hit-matching score of 954/1000 (i.e. extremely high) was obtained by comparing the two spectra. (**B**) 7-point calibration curve of pure D-pantothenic acid standards. For each point, ratios of D-pantothenic acid-peak areas to corresponding internal standard areas (of citric acid-d4) were plotted against the concentrations (µmol) of the analytical-grade D-pantothenic acid standards. For each concentration, synthetic standards were run in duplicate. The excellent linearity observed (R^2=0.9991) demonstrates the stability of D-pantothenic acid in our GC-MS platform. (**C**) Calibration curve used to determine the concentration of D-pantothenic acid in HD and control subjects. A large number of tissue samples were extracted and pooled together to obtain a matrix representative of the subjects and brain regions examined in our study. To obtain a calibration curve, increasing concentrations of analytical-grade D-pantothenic acid standards were added to a pool comprising a mixture of brain-extracts. For each point, ratios of D-pantothenic acid-peak areas to corresponding internal standard areas (citric acid-d4) were plotted against the concentrations of D-pantothenic acid and expressed as µmol/kg brain tissue. On the x-axis, the value 0 corresponds to the endogenous concentration of D-pantothenic acid in the pooled brain extracts. Each concentration in the calibration curve was run in duplicate. The excellent linearity (R^2=0.9951) indicates that our GC-MS assay is well suited to measure D-pantothenic acid in human brain tissue. Abbreviation: IS, internal standard.

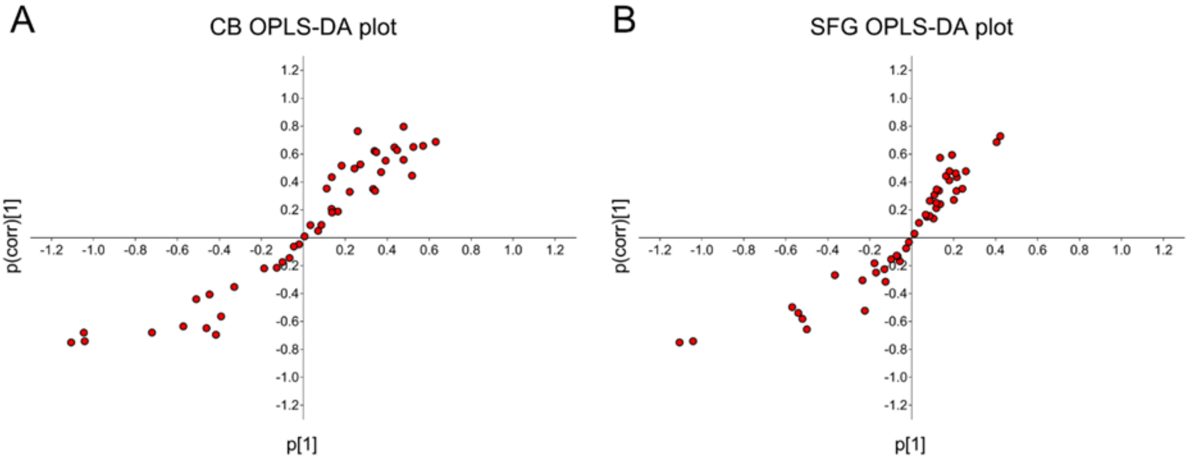

Supplementary Figure S2. S-plots of CB and SFG brain samples were analysed by GC-MS. (**A, B**) For CB and SFG samples, S-plots based on covariance (p[1]) and correlation (p(corr)[1]) of the compounds belonging to the OPLS-DA were used to identify those metabolites contributing the most to the class separation observed in the model. Included in the list of most contributing were several sugars, alternative fuel sources metabolites, urea and vitamin B5

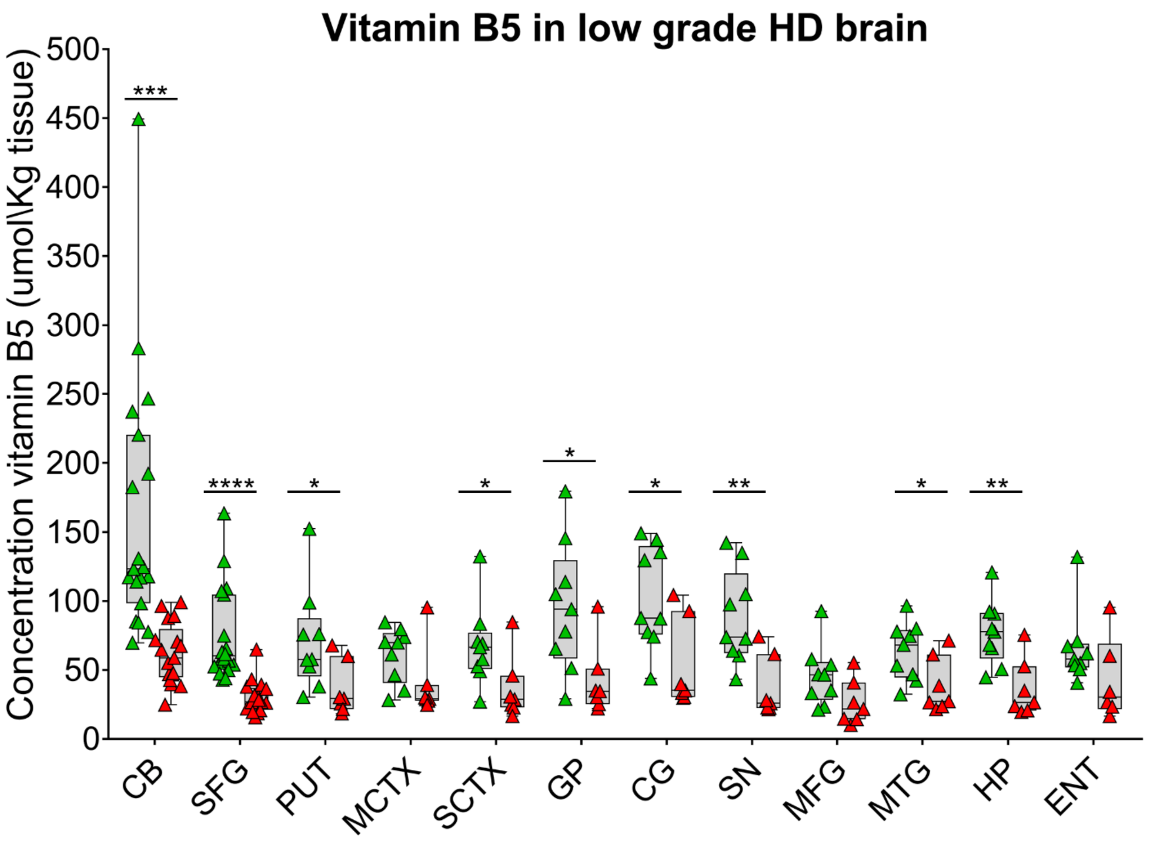

Supplementary Figure S3. Regional distribution of vitamin B5 concentrations in brain tissue illustrating lowered concentrations in cases with HD grades 0-2 compared with controls. Boxplots show distributions of individual vitamin B5 concentrations (µmol/kg tissue) in each of twelve named brain regions of controls (green triangles) and cases with low-grade pathology (red triangles). Vitamin B5 concentrations were significantly decreased in nine out of twelve brain regions examined from these cases with low-grade disease at the time of death, which typically exhibit mild to moderate neuronal loss from affected regions. Multiple *t*-tests were applied for regional case-control contrasts in the 12 functionally-distinct brain regions. Here, the study group comprised only low-grade HD cases (n=18, grades 0-2; red triangles), and all the controls (n = 24; green triangles); boxplots are means ± 95% CI. Abbreviations: *, $p<0.05$; ** <0.005, ***, <0.001; ****, <0.0001.

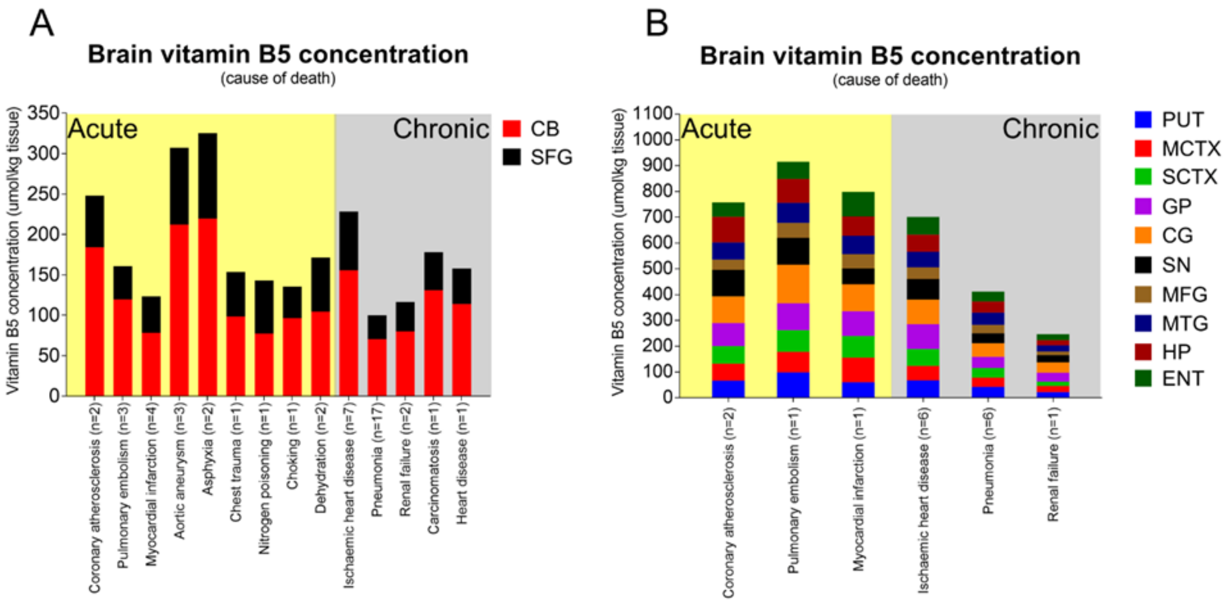

Supplementary Figure S4. Shown is the relationship between causes of death and vitamin B5 concentrations in human brain. (**A, B**) Stacked barplots indicate the contribution that each region has on the cumulative distribution of vitamin B5 in brain (in µmol/kg tissue). The bars are based on individual COD and sub-divided by acute (yellow background) or chronic (grey background) modes of death. The number of subjects (n) available for each COD reported in the study are provided in the x-axis text. COD from all HD cases and controls were included. Abbreviation: COD, cause of death.

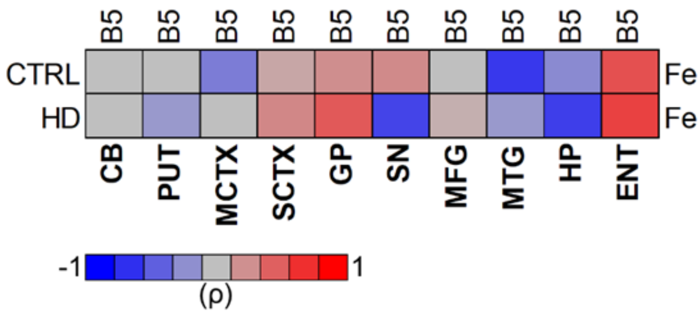

Supplementary Figure S5. Correlation between vitamin B5 and iron in human brain. Heatmap illustrating the correlation of vitamin B5 concentrations (µmol/kg wet tissue) with Fe (µmol/kg dry-weight) in individual brain regions of HD mutation-carriers and matched controls. Colours indicate the degree of correlation of vitamin B5 with the levels of Fe in controls (upper) and cases (lower). Spearman's correlation coefficient (ϱ) values are visually represented by the colour gradient scale in the bottom panel. No significant differences in co-regulation of vitamin B5 and Fe were observed in any of the brain regions examined. The statistical significance of differences in co-regulation between correlations in controls and HD groups was determined by Fisher r-to-z transformation and resulting p-values < 0.05 (two-tailed) were considered significant. Abbreviation: Fe, iron.

© 2019 by the authors. Submitted for possible open access publication under the terms and conditions of the Creative Commons Attribution (CC BY) license (http://creativecommons.org/licenses/by/4.0/).
